# In Vitro Gastrointestinal Digestion Affects the Bioaccessibility of Bioactive Compounds in *Hibiscus sabdariffa* Beverages

**DOI:** 10.3390/molecules28041824

**Published:** 2023-02-15

**Authors:** José de Jesús Rodríguez-Romero, Alejandro Arce-Reynoso, Claudia G. Parra-Torres, Victor M. Zamora-Gasga, Edgar J. Mendivil, Sonia G. Sáyago-Ayerdi

**Affiliations:** 1 Laboratorio Integral de Investigación en Alimentos, Tecnológico Nacional de México/Instituto Tecnológico de Tepic, Av. Tecnológico No 2595, Col. Lagos del Country, Tepic 63175, Mexico; 2 Departamento de Psicología, Educación y Salud, ITESO, Universidad Jesuita de Guadalajara, San Pedro Tlaquepaque 45604, Mexico

**Keywords:** functional beverages, bioactive compounds, phenolic compounds, *Hibiscus sabdariffa*

## Abstract

*Hibiscus sabdariffa* possess great versatility to be used as an ingredient for a whole range of products with natural-based ingredients, which are growing in popularity due to the health benefits of bioactive compounds (BC). Therefore, the objective of this study was to characterize the BC content in *Hibiscus* beverages and to evaluate their in vitro bioaccessibility. Results showed significant differences (*p* < 0.05) in the total contents of BC prior to the in vitro intestinal digestion. Hibiscus acid was the most abundant compound identified. Thirty-five compounds were identified in the *Hibiscus* beverage at the initial stage, while a maximum of 15 compounds were quantified in the different fractions of gastrointestinal digestion. After digestion, significant differences were found compared with the initial content of BC. That phenolic acids were the less bioaccessible group, while flavonoids were the most diverse. Principal components analysis showed different clusters and changes in the profiles of BC present at the initial stage and those bioaccessible, showing that intestinal digestion significantly affects the BC profile of the beverage.

## 1. Introduction

The study of bioactive compounds (BC) in a wide range of food matrices, their functionality, and their beneficial properties have become an elementary aspect of scientific research and innovation in food products. Beverages that include natural-based compounds are growing in popularity due to the enhanced health benefits provided by these ingredients [1]. Most of the time, consumers are unwilling to compromise taste in exchange for health benefits. Therefore, developing new products that effectively bind consumer expectations and preferences is the current challenge for the food and beverage industries.

In Mexico, Hibiscus calyces are mainly used to prepare a refreshing beverage widely consumed by the population, which gives the *H. sabdariffa* crop an economical and cultural importance [2,3]. *Hibiscus* stands out for its characteristic flavor, as well as its potentially beneficial properties to health. It is an essential source of BC, mainly phenolic acids, flavonoids, and anthocyanins [4]. It has been previously reported to have different properties such as diuretic, digestive, anti-inflammatory, and laxative, as well as to moderate hypertension and cholesterol [5,6,7]. However, these compounds must be released during intestinal digestion due to hydrolytic enzymatic activity to be bioaccessible and potentially promote health benefits [8].

Generally, research around *H. sabdariffa* beverages has approached enhancing the phytochemical and physicochemical composition of calyces extracts or optimizing sensory characteristics and consumer acceptance [9,10,11]. Thus, knowledge of the in vitro bioaccessibility of BC after the consumption of *Hibiscus* beverages is currently scarce, and the health effects still need to be fully understood. Within the different varieties of *H. sabdariffa*, an extensive amount of information indicates that selecting a specific variety may determine the outcome of a particular study. Hybrid and improved varieties may present different production yields [2], sensory (such as color and acidity) [12], and BC profiles [5,13,14], affecting the potential functionality of the desired product. It has been previously reported in the study by Duarte-Valenzuela, et al. [15] that improved varieties from different regions of Mexico showed characteristics with functional potential by presenting a higher content of BC and antioxidant activity than the *H. sabdariffa* crops commercially available. Thus, a growing body of evidence on the physical–chemical properties of *H. sabdariffa* has been published in recent years, and approaches to the novel *H. sabdariffa* beverages consumed worldwide are also accumulating [1].

*Hibiscus* extracts containing protocatechuic acid (7%), catechins (9.97%), epigallocatechin (10.23%), epigallocatechin-3-gallate (20%), and caffeic acid (18%) have been shown to possess in vitro antioxidant, nitric oxide inhibitory, and prostaglandin E2 inhibitory activities [16]. Furthermore, the antioxidant capability was improved in diabetic rats after the intake of protocatechuic and gallic acids extracted from *H. sabdariffa* [17].

The mechanisms involved in the anti-inflammatory activities of *H. sabdariffa* extracts appear to be multifunctional, involving different bioactive agents which can interact with different biological targets to elicit the observed anti-inflammatory effects. Recent studies have shown the health effects of aqueous *Hibiscus* extracts [18,19]. Hydroxy citric and Hibiscus are the most abundant organic acids and chlorogenic acids is the main phenolic acid detected in aqueous extracts of *H. sabdariffa*.

Nevertheless, due to the potentially beneficial properties of *H. sabdariffa*, as well as the uprising usage in food preparations and innovation of food products, it is vital to identify the main chemical components and explore how the digestion process impacts these compounds by carrying out studies with registered varieties of *H. sabdariffa*, allowing the development and innovation of functional food products, and suggesting their possible health effects. Hence, increased bioaccessibility of BC is expected from the use of improved *H. Sabdariffa* varieties.

This work aimed to identify and evaluate the bioaccessibility of bioactive compounds by an in vitro digestion model in *H. sabdariffa* beverages.

## 2. Results

### 2.1. Initial Bioactive Compounds in Hibiscus Beverages

Retention time (R_T_), molecular formula, and accurate mass of the quasimolecular ion [M-H]^−^ [M]^+^ after negative and positive ionization by HPLC-ESI-MS in commercial and *Hibiscus* beverage at initial content and after the in vitro gastrointestinal digestion are shown in Table 1. The number assigned to the identified compounds was 1–35, depending on R_T_ and group. Regarding organic acids, trimethylhydroxycitric acid I, hibiscus acid dimethylesther, hydroxycitric acid, hibiscus acid, and trimethylhydroxycitric acid II led this group (IDs: 2–6). Concerning hydroxycinnamic acids and derivatives, ten compounds belong to this group such as caffeoylquinic acid, three isomers of coumaroylquinic acid and caffeoylshikimic acid, chlorogenic acid, and caffeic acid (ID: 8–11 and 13–17) while one hydroxybenzoic acid was identified (ID: 12). Regarding flavonoids, 18 compounds were identified, headed by glycosidic derivatives (ID: 18–32). The presence of compounds derived from flavonoids, such as anthocyanins and anthocyanidins, specifically delphinidin-3-sambubioside, was also found, cyanidin-3-glucoside and delphinidin (ID: 33–35).

The initial content analysis of the extracts revealed the presence of 35 compounds in the *Hibiscus* beverage and 11 compounds in the commercial beverage (Table 2). It is outstanding that the content of BC in *Hibiscus* beverage was three times higher than commercial beverage (417 vs. 138 mg/100 mL).

The presence of organic acids was notable. Hibiscus acid was the most abundant compound identified in both samples. Nevertheless, significant differences (*p* < 0.05) were observed between the samples. The organic acids found in the *Hibiscus* beverage were significantly higher in concentration compared to those in the commercial beverage, as shown in Table 2. In the commercial beverage, hibiscus acid accounted 70.2 mg/100 mL, while for the *Hibiscus* beverage had 231.52 mg/100 mL. Since hibiscus acid is the predominant compound in the commercial beverage, this translated into a higher acidity than the *Hibiscus* beverage, which had repercussions in consumers’ acceptance.

Regarding hydroxycinnamic acids and their derivatives, caffeoylquinic acids and isomers of coumaroylquinic and caffeoylshikimic acids were predominant in *Hibiscus* beverage (up to 51.38 mg/100 mL). In contrast, only caffeoylquinic acid, coumaroylquinic acid II, and chlorogenic acid were detected in minor amounts in the commercial beverage (7.84 mg/100 mL). As previously reported, *H. sabdariffa* has been considered a rich source of hydroxycinnamic acids and derivatives [4,5] and considering that the *Hibiscus* beverage contains mint, Eftekhari, et al. [20] reported that hydroxycinnamic acids and its derivatives are commonly found in the genus *Mentha*.

Up to 18 flavonoids were quantified at the initial stage in the *Hibiscus* beverage, whereas in contrast, only three flavonoids were identified in the commercial beverage (Table 2). Glycosidic derivatives of flavonols (quercetin and kaempferol), aglycones (luteolin, quercetin, myricetin, and naringenin) were quantified in the beverage. These compounds are in concordance with the reported data by several studies which previously identified and quantified these compounds in *Hibiscus* and mint [4,5,10,21].

It has been previously stated that phenolic compounds are associated to astringency and bitter taste in foods and beverages. Hydroxycinnamic acids and their derivatives, as well as flavonol glycosides found in *Hibiscus* extracts, have been reported to have an astringency taste in red wines [1,22]. This intense flavor is counterbalanced by adding sweeteners, improving consumer preference [1].

Table 2 showed significant differences (*p* < 0.05) in the total contents of BC prior to the in vitro intestinal digestion. The commercial beverage accounted for 138.05 mg/100 mL, and this total initial content of bioactive compounds was significantly lower (*p* < 0.05) than the compounds found in the beverage (417.78 mg/100 mL). It must be pointed out that this difference is mainly due to the content of hibiscus acid, which as previously stated, affected consumers acceptance of the product. Moreover, the *Hibiscus* beverage is free of synthetic additives (i.e., food flavoring or colorants). Thus, their initial bioactive compounds profile found is explained by the mixture of natural ingredients such as *Hibiscus* and mint. Moreover, the *Hibiscus* extract used in this preparation comes from specific varieties of *Hibiscus sabdariffa* L. found in Mexico, which have reported a higher and more diverse content of BC [14]. Borrás-Linares et al. [16] reported that the extracts of PC and flavonoids from *Hibiscus* could be used for the development of functional food and nutraceuticals. However, the analysis of the bioaccessibility of these compounds is also required [5].

### 2.2. Bioactive Compounds Released during In Vitro Intestinal Digestion

A critical topic around the optimization of the production and formulation of novel functional beverages is the study of the interactions that might occur among the food matrix components (mainly bioactive compounds-fiber-protein-lipids) of the beverage after the mixture of the ingredients used for its production. One of the challenges in the functional beverages industry is to accomplish the “optimal content high enough” of each BC to exert health benefits [23]. Furthermore, it is essential to evaluate the bioaccessibility of the BC to prove beverage functionality effectively. In light of this, several steps have been reported to design a functional beverage, including identifying and quantifying promising bioactive compounds, researching their bioaccessibility, bioavailability, and metabolism, and the potential interactions among the components of the food matrix [22].

Seventeen compounds were identified in the intestinal digestion fraction, with compounds below the limit of quantification (Table 3). A higher content of organic acids and related compounds was observed in the commercial beverage, not in the *Hibiscus* beverage with significant difference (*p* > 0.05). After centrifugation of the supernatants on gastrointestinal digestion in the soluble indigestible fraction, only seven compounds were quantified related to the organic acids, all the other BC were not detected, besides the *Hibiscus* beverage were caffeoylquinic acid and ellagic acid remained in the beverage; however, the content of BC in the Hibiscus beverage was lower than commercial sample, this indicates that most of the compounds quantified before were potentially bioaccesible. In the insoluble indigestible fraction, the commercially beverage the organic acids remain in the residue, indicating that other compounds in this beverage are able to link this compounds and reduce their bioaccessibility. In the case of Hibiscus beverage, the insoluble fraction was lower, this indicates that most of the compounds are bioaccesible (Table 3). Hibiscus acid was the most bioaccessible compound in the *Hibiscus* beverage, and a decrease in concentration was observed compared with the initial content. Bioaccessibility is also influenced by the decoction process used in beverage preparation, as reported by Mercado-Mercado et al. [24].

### 2.3. Initial Content and Indigestible Fractions after Gastrointestinal Digestion by Groups: Multivariate Data Analysis

PCA was performed on BC concentrations to address patterns among the profiles of BC found in samples at initial content and those non-bioaccessible after gastrointestinal digestion by groups (phenolic acids and flavonoids). For each group, two principal components (PCs) were obtained (Eigenvalues > 1) that explained over 72.7 and 83.3% respectively of the total variance among the samples. PCA analysis showed differentiated BC profile patterns at the different stages evaluated between the beverages. The compounds located in the axis of the PCs at the stage evaluated were based on factors coordinated values <−1.0 for less influence (negative axis) and >1.0 for higher influence (positive axis).

**Figure 1:**
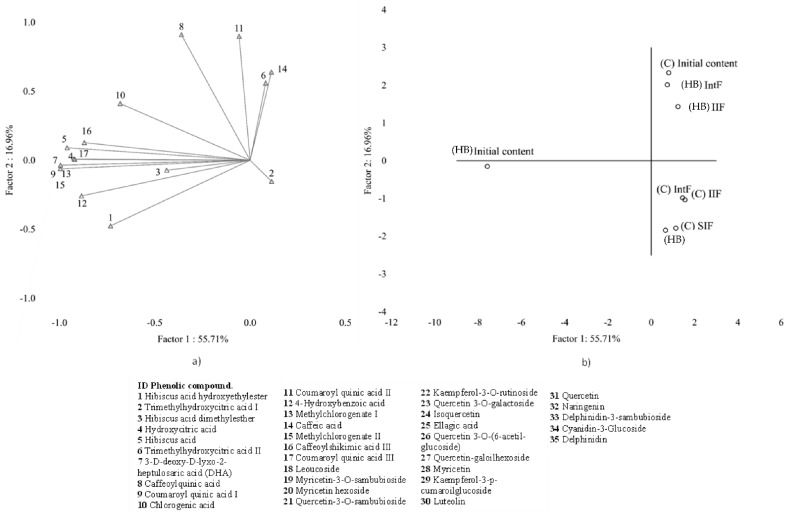
Analysis of phenolic acids patterns by group found in the extracts from “initial content” and during in vitro gastrointestinal digestion of commercial (C) and *Hibiscus* beverage (HB) using principal component analysis (PCA): (**a**) Projection of the variables on the factor-plane, (**b**) projection of the cases on the factor-plane.

**Figure 2:**
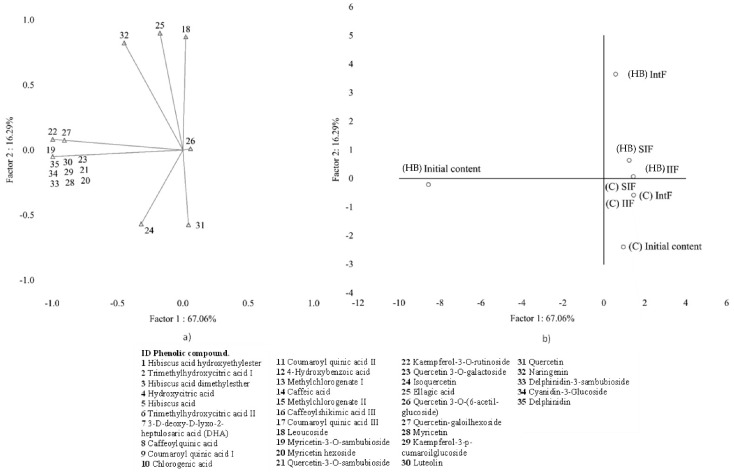
Analysis of flavonoids patterns by group found in the extracts from “initial content” and during in vitro gastrointestinal digestion of commercial (C) and *Hibiscus* beverage (HB) using principal component analysis (PCA): (**a**) Projection of the variables on the factor-plane, (**b**) projection of the cases on the factor-plane.

Figure 1a and Figure 2a show a projection of the variables on the factor-plane for the two principal components, showing change in the BC profiles by group. Figure 1a shows the PCA for the group of phenolic acids. It revealed that the differences observed were derived mainly from the influence of compounds found in the initial stage of the *hibiscus* beverage, corresponding to the PC1 negative axis (ID: 1, 3, 4, 5, 7, 9, 12–17). PC1 of both groups corroborates Table 3 data because this pattern represents that the *Hibiscus* beverage has more bioaccessible BC compared to the commercial beverage.

PC2 on the positive axis was highly influenced by glycosides such as leucoside, and aglycones such as ellagic acid, and naringenin (ID: 18, 25, 32). On the other hand, PC1 negative axis comprises compounds that were quantified at relatively low levels (ID: 19, 20, 22, 28, 29, 30, 33, 34, 35). However, these compounds improve the profile of BC in the *hibiscus* beverage.

Furthermore, Figure 1b and Figure 2b show that the samples are divided into three well-defined clusters when the profile of BC at the initial stage and intestinal digestion fractions are compared among all the samples. The different clusters showed no correlation between the profile of the bioactive compounds at the initial stage and those bioaccessible since the *Hibiscus* beverage initial content profile was located in PC1 negative axis. In contrast, its bioaccessible profile changed and was located in both groups’ PC1 and PC2 positive axis. However, it must also be remarked that in the commercial beverage, no significant compounds were associated with this group. Thus, the *Hibiscus* beverage may provide a significant amount of BC potentially bioaccessible. Studies revealed that the profile of BC at the initial stage differs from those released in intestinal digestion [25], confirming the results found in this work.

## 3. Discussion

After gastrointestinal digestion, the flavonoids and related compounds group found in the indigestible fractions comprise aglycones and glycosidic derivatives. Aglycones such as quercetin and derivatives, such as quercetin-galloylhexoside and Quercetin 3-*O*-(6-acetil-glucoside), ellagic acid, and myricetin were quantified in the *Hibiscus* beverage (Table 3). Ellagic acid was recently found as bioaccessible in guava (*P. guajava* L. ‘Amarilla’), this is related to the digestion process, where the enzymes could release ellagic acid from ellagitannin structure linked to the cell wall [26]. Even though ellagic acid may be bioaccessible in the small intestine, its bioavailability is still unclear. Thus it may be more likely to be used as a substrate for the gut microbiota during colonic fermentation [27]. It is suggested that the digestion process (enzymes, pH changes) may increase the release of the bound phenolic compounds and those interacting with the components of the food matrix, mainly dietary fiber [28]. In this context, as reported by Sáyago-Ayerdi et al. [4], phenolic compounds (that interact with dietary fiber or resist the gastrointestinal digestion) from *Hibiscus* may reach the colon, becoming available substrate for the gut microbiota, which may result as beneficial to the health.

**Figure 3:**
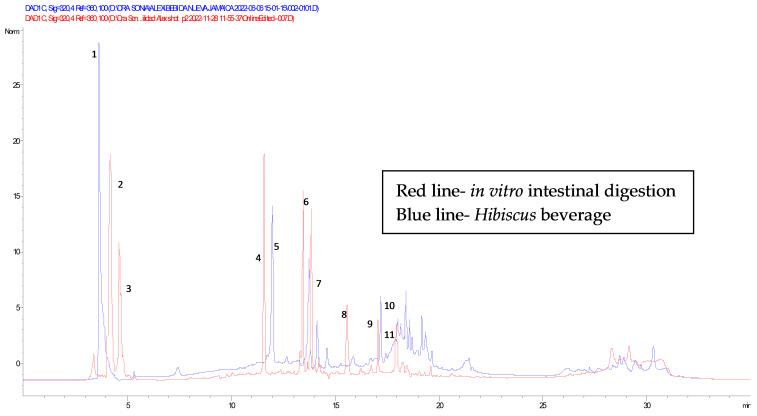
Chromatograms of bioactive compounds (BC) identified by HPLC-DAD of the Hibiscus beverage in the initial phase, and chromatogram of BC released during in vitro intestinal digestion. CB detected: 1–2 hibiscus acid, 3 hydroxycitric acid, 4–5 caffeoylquinic acid, 6–7 chlorogenic acid, 8 caffeic acid, 9–10 caffeoylshikimic acid III, 11 quercetin-galloylhexoside.

One aspect to consider is that those phenolic compounds released during gastrointestinal digestion may also reach the bloodstream and have health benefits. In this context, the significant reduction in BC found from the initial content in the *hibiscus* beverage (285.24 mg/100 mL) to the different intestinal fractions (26.70 mg/100 mL) corresponding to a reduction of approximately 90% is probably due to the above-mentioned reason. This behavior is shown in Figure 3.

In light of this, Rasheed, et al. [29], reported that the BC profile from the initial content of *Hibiscus* hot and cold beverages was found to have a reasonable correlation for the inhibition of α-glucosidase enzyme, and its regulation is linked to the prevention of type II diabetes. Nevertheless, the BC must be released from the food matrix to present this activity.

**Figure 4:**
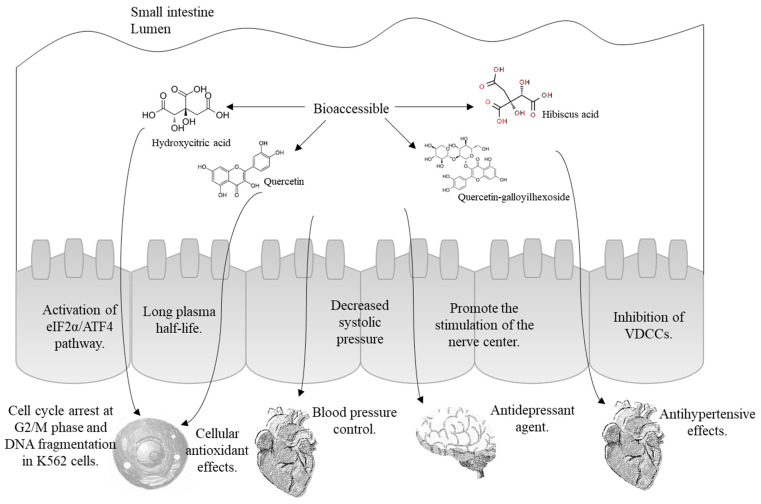
Potential health effects of the main bioactive compounds from the *Hibiscus* beverage.

Figure 4 highlights the possible effects that the compounds found in the *Hibiscus* beverage (shown in Table 2 and Table 3) may exert if they are absorbed into the bloodstream. An important mechanism involved in the anti-inflammatory activities of hibiscus extracts is its ability to suppress the generation of oxidative stress and cellular damage in cells. The conjugated forms of quercetin and kaempferol often detected in the plasma after intake of hibiscus could indicate long-lasting cellular antioxidant effects because of their long plasma half-life [30]. Recently, hibiscus acid was reported as a nutraceutical approach for the treatment of chronic myelogenous leukemia due to protein response observed by activation of eIF2α/ATF4 pathway that induced cell cycle arrest at G2/M phase and DNA fragmentation in leukemia K562 cells [31]. There is considerable evidence in the literature showing that BC found in *Hibiscus sabdariffa* has an antihypertensive effect, which is further supported by a number of in vitro studies demonstrating a vasorelaxant effect of the crude extract of this plant [32]. Hibiscus acid has a direct vasorelaxant effect on the rat aorta, through the inhibition of VDCCs (voltage-dependent calcium channels), inhibiting the influx of extracellular Ca^2+^ (a component of the contractile response). Then, this compound may be the constituent responsible for the vascular activity of this plant [33]. It was also found in a recent study that hibiscus acid presents anti-microbial activity against some pathogenic bacteria, concluding that it was one of the compounds responsible for the anti-microbial effect of *Hibiscus* calyxes [34].

Regarding flavonoids, it has been reported that quercetin-3-sambubioside could be used as a potential antidepressant agent because it promoted the stimulation of the nerve center according to the evaluation of convulsion rate in mice [35]. Related to this, it was also reported that quercetin decreased blood pressure in normotensive and (pre)hypertensive patients showing promising antihypertensive effects [36].

## 4. Materials and Methods

### 4.1. Reagents

The chemicals, enzymes [pepsin (P-7000), pancreatin (P-1750) and α-amylase (A-6255)] and analytical standards [gallic acid (G7384-100G), naringenin (N5893-1G), ellagic acid (E2250-1G), catechin (C1251-5G), myricetin (70050-25MG), quercetin (Q4951-10G), kaempferol (K0133-10MG), gallocatechin gallate (G6782-5MG), caffeic acid (C0625-2G), vanillic acid (68654-50MG), trans-cinnamic acid (C80857-5G), chlorogenic acid (C3878-250MG), p-coumaric acid (C9008-1G), ferulic acid (46278-1G-F) and garcinia acid (44292-10 mg)] were provided by Sigma-Aldrich (St. Louis, MO, United States).

### 4.2. Sample Preparation

About 60 mL of filtered *H. sabdariffa* beverage was prepared with a mixture of hibiscus varieties cultivated from the region of Jala, Nayarit (21° 39′ 15″ N, 106° 32′ 45″ O), Mexico. A decoction with the hibiscus calyces was carried out for 5–6 min at an 80–90 °C temperature, and centrifuged at 700–1100 rpm. The temperature was lowered to 60–70 °C and finally citric acid, mint (added at the beginning of the preparation of the beverage from dried crushed mint leaves), and stevia as a sweetener (97% of purity of rebaudioside A, Metco, Mexico DF, Mexico) were added and all ingredients were homogenized for 5–10 min and cooled to room temperature, according to the patent MX/a/2022/010704. Samples were stored at room temperature (25 °C), and the commercial *Hibiscus sabdariffa* beverage used as control was purchased from a nutritional store sold as an antioxidant beverage.

### 4.3. Bioaccessibility of Phenolic Compounds after In-Vitro Gastrointestinal Digestion

A *Hibiscus* beverage and an *Hibiscus* commercial drink as the control beverage were subjected to a static in vitro gastrointestinal digestion model according to Blancas-Benitez, et al. [37] to evaluate the bioaacessibility of phenolic compounds (PC). Gastric digestion was simulated by adding pepsin (P-7000, Sigma-Aldrich, 0.2 mL of a 300 mg/mL solution in 0.2 M HCl-KCl buffer, pH 1.5, 40 °C, 2 h). Pancreatin (P-1750, Sigma-Aldrich, 3 mL of a 5 mg/mL solution in 0.1 M phosphate buffer, pH 7.5, 37 °C, 2 h) was added in order to simulate the intestinal digestion. After the intestinal digestion, this fraction was used to evaluated the BC released. This method differs from other methods such as the INFOGEST protocol [38], where a dialysis bag is used for the simulation of the passive diffusivity of metabolites in the small intestine; in this study, sample dialysis bags were not used because of the type of sample. The samples from this stage were centrifuged (Hermle Z 323 K; Wehingen, Germany) (3500× *g*, 15 min, 4 °C), and the supernatant (digested extract) was considered as soluble indigestible fraction. The residue was considered as indigestible-fraction. Both fractions were used to identify the BC by HPLC-DAD-ESI-MS described in the section below.

### 4.4. Determination of Phenolic Compounds and Organic Acids Profile by HPLC-DAD-ESI-MS

An aqueous-organic extraction was performed on the *Hibiscus* beverage as well as the commercial beverage [39] named as initial stage, and to the supernatant from digested fractions considered as intestinal fraction, soluble indigestible fraction, and residue considered as, insoluble indigestible fraction; all these samples were identified by HPLC-DAD-ESI-MS. These supernatants from aqueous-organic extraction were dispensed in microtubes (Eppendorf, Hamburg, Germany) (2 mL), centrifuged (Vacufuge plus, Eppendorf) (14,000 rpm, 20 min), and filtered through a 0.45 µm nylon membrane filter (Merck Millipore Ltd., Cork, Ireland) and dispensed in chromatographic vials.

The identification of PC by HPLC-DAD-ESI-MS was carried out according to Blancas-Benitez, Pérez-Jiménez, Montalvo-González, González-Aguilar and Sáyago-Ayerdi [38]. An HPLC Agilent 1260 series system (Agilent Technologies, Santa Clara, CA, USA) equipped with an Agilent G4212-60008 UV-Vis diode array detector (DAD) and coupled with a 6120 Agilent simple Quadrupole LC/MS with an electrospray ionization interface in negative and positive ionization mode (N_2_ as drying gas flow, 13.0 L/min; nebulizer pressure, 40 psi; gas drying temperature, 350 °C; capillary voltage, 3500 V). A volume sample of 10 µL was automatically injected (flow rate 0.4 mL/min) onto a Poroshell 120 EC-C18 column (4.6 mm × 150 mm, particle size 2.7 µm) (Agilent Technologies). The elution gradient was prepared using water containing 0.1 % formic acid as solvent A and acetonitrile as solvent B (Sigma Aldrich). The data analysis was performed using OpenLab CDS, ChemStation Edition software (Agilent Technologies). Characterization of the BC was based on retention time (R_T_) in DAD and mass spectrometric signal (single MS scan in the 100–1000 m/z range) directly compared with the R_T_ of analytical standards and also based on previously m/z ions from *Hibiscus* described in previous studies [4]. External calibration curves were prepared to quantify the compounds identified. When the standard was unavailable, the calibration curve corresponding to its phenolic precursor was used to quantify the compound (Table A1).

### 4.5. Statistical Analysis

All analyses were performed in triplicate; each determination was used to calculate the mean values and standard deviations. Data were analyzed by ANOVA/Fisher’s test for all test samples (*p* < 0.05, *n* = 3). Datasets of BC profiles were evaluated between samples and intestinal digestion or initial stage using principal components analysis (PCA). Each data set was treated individually, and then compared the patterns displayed by groups of the initial content of CB vs. the content after in vitro digestion of the *Hibiscus* and the commercial beverage.

All analyses were performed using STATISTICA software, version 10.0 (Stat Soft. Inc. 1984-2007, Tulsa, OK, USA).

## 5. Conclusions

Innovation and development of food products with a potential benefit to human health require research related to how bioactive compounds behave in the digestion process, and the data obtained in this work showed that the beverage formulated with *H. sabdariffa* and mint contains 35 bioactive compounds, in comparison with the 11 from an already market-available product, highlighting the use of *Hibiscus* in a shot beverage form. The principal component analysis demonstrated that the digestion process significantly affects the bioactive compound profile that may benefit health, distinguishing differentiated groups among the beverages with an increased bioaccessibility of phenolic acids and flavonoids. However, the generation of knowledge related to this process must be carried out comprehensively, and further in vivo studies that evaluate the bioavailability of these bioactive compounds must be taken into account besides the organoleptic and flavor properties when developing a functional food product with such gastronomical importance as *H. sabdariffa.*

## 6. Patents

82716. Bebida de Jamaica con alta concentración de antioxidantes y fibra dietética y proceso de obtención de la misma. MX/a/2022/010704.

## Figures and Tables

**Table 1:** Phenolic compounds and organic acids identified by HPLC-DAD-ESI-MS in commercially and *Hibiscus* beverages at the initial stage and released during in vitro intestinal digestion.

ID	Tentative Compound	R_T_ (min)	Molecular Formula	[M-H]^−^ or [M]^+^
Organic acids and related compounds				
1	Hibiscus acid hydroxyethyl ester	3.07	C_8_H_12_O_8_	235
2	Trimethylhydroxycitric acid I	3.12	C_9_H_14_O_8_	249
3	Hibiscus acid dimethylesther	3.55	C_8_H_10_O_7_	217
4	Hydroxycitric acid	3.83	C_6_H_8_O_8_	207
5	Hibiscus acid	4.10	C_6_H_6_O_7_	189
6	Trimethylhydroxycitric acid II	4.85	C_9_H_14_O_8_	249
7	3-D-deoxy-D-lyxo-2-heptulosaric acid (DHA)	4.86	C_7_H_10_O_8_	221
Phenolic acids and related compounds				
8	Caffeoylquinic acid	11.93	C_16_H_18_O_9_	353
9	Coumaroyl quinic acid I	13.64	C_16_H_18_O_8_	337
10	Chlorogenic acid	13.75	C_16_H_18_O_9_	353
11	Coumaroyl quinic acid II	15.84	C_16_H_18_O_8_	337
12	4-Hydroxybenzoic acid	14.57	C_7_H_6_O_3_	138
13	Methylchlorogenate I	15.00	C_17_H_20_O_9_	367
14	Caffeic acid	15.89	C_9_H_8_O_4_	179
15	Methylchlorogenate II	16.50	C_17_H_20_O_9_	367
16	Caffeoylshikimic acid III	17.49	C_16_H_16_O_8_	335
17	Coumaroyl quinic acid III	20.85	C_16_H_18_O_8_	337
Flavonoids and related compounds				
18	Leoucoside	13.49	C_26_H_28_O_15_	579
19	Myricetin-3-*O*-sambubioside	15.51	C_26_H_28_O_17_	612
20	Myricetin hexoside	16.53	C_21_H_20_O_13_	480
21	Quercetin-3-*O*-sambubioside	16.97	C_26_H_28_O_16_	596
22	Kaempferol-3-*O*-rutinoside	17.24	C_27_H_30_O_15_	594
23	Quercetin 3-*O*-galactoside	17.49	C_21_H_20_O_12_	464
24	Isoquercetin	17.74	C_21_H_20_O_12_	463
25	Ellagic acid	17.83	C_14_H_6_O_8_	301
26	Quercetin 3-*O*-(6-acetil-glucoside)	17.85	C_21_H_20_O_12_	506
27	Quercetin-galoilhexoside	17.97	C_21_H_20_O_12_	616
28	Myricetin	19.53	C_15_H_10_O_8_	317
29	Kaempferol-3-p-cumaroilglucoside	19.93	C_30_H_26_O_13_	594
30	Luteolin	20.34	C_15_H_10_O_6_	286
31	Quercetin	20.80	C_15_H_10_O_7_	301
32	Naringenin	21.38	C_15_H_12_O_5_	272
Anthocyanins and anthocyanidins				
33	Delphinidin-3-sambubioside	11.90	C_26_H_29_O_16_+	598
34	Cyanidin-3-Glucoside	12.32	C_15_H_11_O_7_-	302
35	Delphinidin	13.63	C_21_H_21_O_11_+	550

R_T_ = retention time (min).

**Table 2:** The initial content of phenolic compounds and organic acids present in commercially available *Hibiscus* beverages.

			Beverages	
ID	Tentative Compound	R_T_ (min)	Commercial	*Hibiscus* Beverage (HB)
Organic acids and related compounds				
1	Hibiscus acid hydroxyethyl ester	3.07	n.d.	1.85 ± 1.07
2	Trimethylhydroxycitric acid I	3.12	1.31 ± 0.71 ^a^	0.51 ± 0.53 ^a^
3	Hibiscus acid dimethylesther	3.55	18.33 ± 0.4 ^a^	18.31 ± 5.36 ^a^
4	Hydroxycitric acid	3.83	7.21 ± 2.11 ^a^	31.25 ± 5.68 ^b^
5	Hibiscus acid	4.10	70.20 ± 11.50 ^a^	231.52 ± 50.96 ^b^
6	Trimethylhydroxycitric acid II	4.85	28.69 ± 0.84 ^a^	0.74 ± 0.07 ^b^
7	3-D-deoxy-D-lyxo-2-heptulosaric acid (DHA)	4.86	n.d.	1.06 ± 0.40
Total organic acids and related compounds mg/100 mL			125.74 ± 22.0 ^a^	285.24 ± 54.15 ^b^
Phenolic acids and hydroxycinnamic acids derivatives				
8	Caffeoylquinic acid	11.93	7.09 ± 0.66 ^a^	6.59 ± 1.43 ^a^
9	Coumaroyl quinic acid I	13.64	n.d.	0.52 ± 0.15
10	Chlorogenic acid	13.75	0.21 ± 0.41 ^a^	3.36 ± 2.24 ^a^
11	Coumaroyl quinic acid II	15.84	0.54 ± 0.15 ^a^	0.15 ± 0.13 ^a^
12	4-Hydroxybenzoic acid	14.57	n.d.	0.18 ± 0.31
13	Methylchlorogenate I	15.00	n.d.	0.97 ± 1.69
14	Caffeic acid	15.89	n.d.	0.42 ± 0.38
15	Methylchlorogenate II	16.50	n.d.	38.55 ± 23.66n
16	Caffeoylshikimic acid III	17.49	n.d.	0.44 ± 0.10
17	Coumaroyl quinic acid III	20.85	n.d.	0.19 ± 0.19
Total phenolic acids and related compounds mg/100 mL			7.84 ± 1.72 ^a^	51.38 ± 26.10 ^b^
Flavonoids and related compounds				
18	Leoucoside	13.49	n.d.	0.04 ± 0.03
19	Myricetin-3-*O*-sambubioside	15.51	n.d.	5.40 ± 7.40
20	Myricetin hexoside	16.53	n.d.	1.01 ± 0.00
21	Quercetin-3-*O*-sambubioside	16.97	n.d.	4.78 ± 0.00
22	Kaempferol-3-*O*-rutinoside	17.24	n.d.	0.79 ± 0.00
23	Quercetin 3-*O*-galactoside	17.49	n.d.	0.04 ± 0.00
24	Isoquercetin	17.74	0.23 ± 0.07 ^a^	0.10 ± 0.11 ^a^
25	Ellagic acid	17.83	n.d.	0.98 ± 0.30
26	Quercetin 3-*O*-(6-acetil-glucoside)	17.85	n.d.	0.01 ± 0.00
27	Quercetin-galloylhexoside	17.97	2.76 ± 2.07 ^a^	7.03 ± 0.01 ^b^
28	Myricetin	19.53	n.d.	0.30 ± 0.43
29	Kaempferol-3-p-cumaroilglucoside	19.93	n.d.	0.13 ± 0.00
30	Luteolin	20.34	n.d.	0.01 ± 0.00
31	Quercetin	20.80	1.48 ± 0.59 ^a^	0.10 ± 0.04 ^b^
32	Naringenin	21.38	n.d.	0.03 ± 0.00
Total flavonoids and related compounds mg/100 ml			4.47 ± 0.90 ^a^	20.75 ± 0.54 ^b^
Anthocyanins and anthocyanidins				
33	Delphinidin-3-sambubioside	11.90	n.d.	39.88 ± 37.29 ^a^
34	Cyanidin-3-glucoside	12.32	n.d.	14.05 ± 12.40 ^a^
35	Delphinidin	13.63	n.d.	6.48 ± 3.87 ^a^
Total anthocyanins and anthocyanidins mg/100 mL			n.d.	60.41 ± 53.21 ^a^
TOTAL (mg/100 mL)			138.05 ± 50.24 ^a^	417.78 ± 45.85 ^b^

Values represent mean ± SD (*n* = 3). Different lowercase letters in the same row indicate significant differences between beverages (*p* < 0.05). n.d. = Not detected.

**Table 3:** Phenolic compounds and organic acids content after an in vitro gastrointestinal digestion of commercial (CB) and *Hibiscus* beverage (HB).

		Intestinal Fraction		Soluble Indigestible Fraction		Insoluble Indigestible Fraction	
ID	Tentative Compound	CB	HB	CB	HB	CB	HB
Organic acids and related compounds							
1	Hibiscus acid hydroxyethyl ester	n.d.	<LOQ	1.29 ± 0.31 ^a^	1.08 ± 0.12 ^a^	n.d.	n.d.
2	Trimethylhydroxycitric acid I	n.d.	1.14 ± 0.49	1.93 ± 0.69 ^a^	2.07 ± 0.29 ^a^	n.d.	0.33 ± 0.18
3	Hibiscus acid dimethylesther	10.56 ± 5.06 ^a^	2.58 ± 0.55 ^b^	19.46 ± 2.64 ^a^	3.77 ± 0.71 ^b^	n.d.	n.d.
4	Hydroxycitric acid	n.d.	n.d.	n.d.	1.48 ± 1.06	10.61 ± 1.87 ^a^	1.20 ± 0.61 ^b^
5	Hibiscus acid	16.00 ± 3.56 ^a^	3.37 ± 2.79 ^b^	2.20 ± 0.59 ^a^	9.16 ± 2.29 ^b^	13.33 ± 6.94	n.d.
6	Trimethylhydroxycitric acid II	n.d.	<LOQ	n.d.	<LOQ	n.d.	n.d.
Total organic acids and related compounds		26.56 ± 5.06 ^a^	7.50 ± 1.17 ^b^	24.88 ± 3.50 ^a^	17.67 ± 2.45 ^a^	23.94 ± 6.70 ^a^	1.53 ± 0.79 ^b^
Hydroxycinnamic acids and related compounds							
8	Caffeoylquinic acid	n.d.	7.90 ± 1.28	n.d.	0.13 ± 0.02	n.d.	6.99 ± 4.04
9	Coumaroyl quinic acid III	n.d.	<LOD	n.d.	<LOQ	n.d.	0.22 ± 0.12
10	Chlorogenic acid	n.d.	3.40 ± 1.07	n.d.	n.d.	n.d.	n.d.
13	Coumaroyl quinic acid II	n.d.	0.23 ± 0.03	n.d.	n.d.	n.d.	0.23 ± 0.17
14	Caffeic acid	n.d.	4.22 ± 0.85	n.d.	n.d.	n.d.	3.49 ± 2.02
Total hydroxycinnamic acids and related compounds		-	15.83 ± 3.34 ^b^	-	0.13 ± 0.02	-	10.93 ± 6.31
Flavonoids and related compounds							
18	Leoucoside	n.d.	0.74 ± 0.06	n.d.	<LOQ	n.d.	n.d.
28	Quercetin-galloylhexoside	n.d.	2.83 ± 0.06	n.d.	<LOQ	n.d.	n.d.
26	Ellagic acid	n.d.	2.45 ± 0.20	n.d.	1.29 ± 0.28	n.d.	1.07 ± 0.13
23	Kaempferol-3-rutinoside	n.d.	0.12 ± 0.03	n.d.	<LOQ	n.d.	n.d.
27	Quercetin 3-*O*-(6-acetil-glucoside)	<LOQ	Nd	<LOQ	n.d.	<LOQ	<LOQ
32	Naringenin	n.d.	<LOQ	n.d.	n.d.	n.d.	n.d.
Total flavonoids and related compounds		-	6.26 ± 0.16	-	1.29 ± 0.28	-	
	TOTAL (mg/100 mL)	26.56 ± 1.06 ^a^	29.59 ± 1.81 ^a^	24.88 ± 1.05 ^a^	19.40 ± 2.2 ^a^	23.94 ± 6.70 ^a^	13.65 ± 8.32 ^b^

Values represent mean ± SD (*n* = 3). Different lowercase letters in the same row indicate a significant difference (*p* < 0.05) between beverages for each fraction. (<LOQ) = below the limit of quantification. n.d. = not detected. HB = Hibiscus Beverage.

## Data Availability

Data is contained within the article.

## Appendix A

**Table A1:** Analytical parameters of the method of quantification of bioactive compounds.

Standard	Equation	R^2^	LOQ ^1^ (μM/mL)
kaempferol	y = 24766x + 230022	0.9939	3.26
*p*-coumaric acid	y = 90663x + 16308	0.9996	1.14
Naringenin	y = 210592x + 130168	0.9965	3.34
Garcinia acid	y = 7429.8x +24963	0.9979	1.00
Quercetin	y = 1324209x + 78664	0.9979	2.58

^1^ LOQ, limits of quantification.
